# Supplementary material for: Structural polymorphism and substrate promiscuity of a ribosome-associated molecular chaperone
Source: Magn Reson (Gott). 2021 Jun 4;2(1):375–86. doi: 10.5194/mr-2-375-2021 (PMC10539794; doi:10.5194/mr-2-375-2021)
Supplement: The supplement related to this article is available online at: https://doi.org/10.5194/mr-2-375-2021-supplement. [file mr-2-375-supplement.pdf]

Supplement of Magn. Reson., 2, 375–386, 2021
https://doi.org/10.5194/mr-2-375-2021-supplement
© Author(s) 2021. CC BY 4.0 License.

Supplement of

Structural polymorphism and substrate promiscuity of a ribosome-associated molecular chaperone

Chih-Ting Huang et al.

Correspondence to: Shang-Te Danny Hsu (sthsu@gate.sinica.edu.tw)

The copyright of individual parts of the supplement might differ from the article licence.

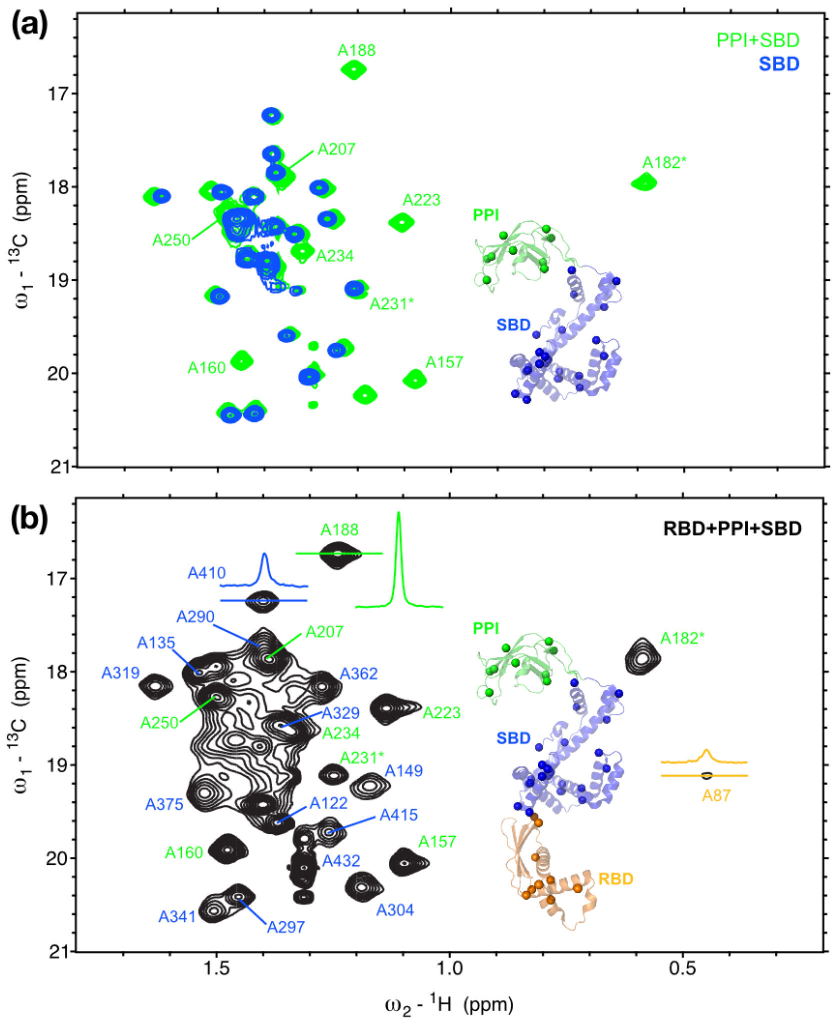

Figure S1. Alanine methyl NMR spectral analyses of TF variants. (a) Superposition of the methyl ^{13}C-^{1}H correlation spectra of SBD (blue) and PPI+SBD (green) with selected crosspeaks originated from PPI labeled with their residue identities. (b) Methyl ^{13}C-^{1}H correlation spectra of full-length TF with resolved crosspeaks labeled with the corresponding residue identities. The proton lineshapes of selected resonances are shown for RBD, PPI and SBD as indicated by the horizontal lines. Insets: cartoon representations of PPI+SBD (a) and full-length TF (b) with RBD, PPI and SBD colored in orange, green and blue, respectively. The Cβ atoms of the selectively protonated and ^{13}C labeled alanine side-chains are shown in spheres. All constructs were perdeuterated with selective protonation and ^{13}C labeling at the alanine Cβ positions.

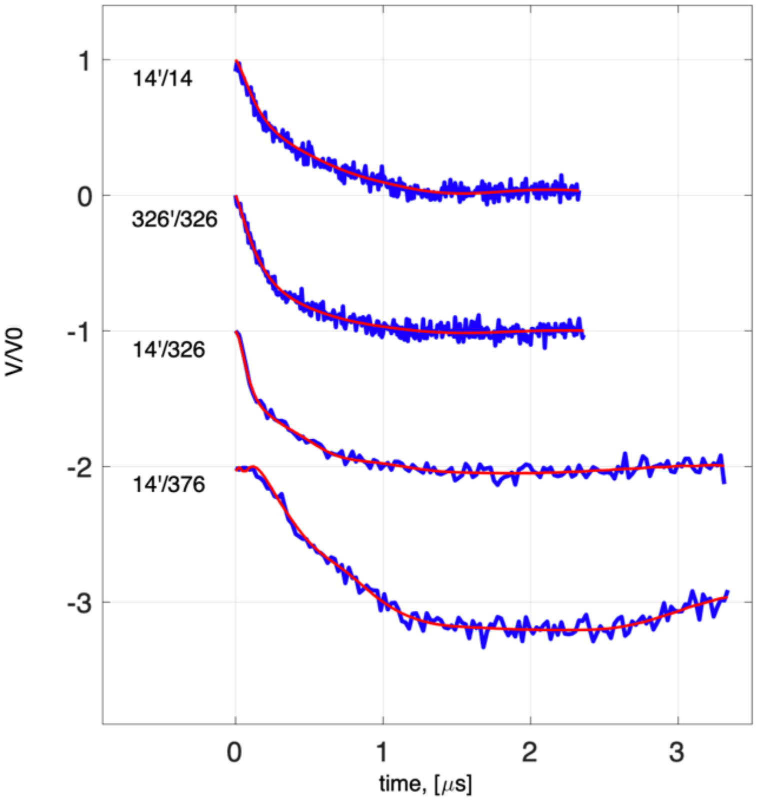

Figure S2. DEER data of singly labeled TF variants mixture. The time-domain DEER traces (after the background correction) are shown by blue lines with y-axis shift. Simulated traces recovered from the Tikhonov-based results are shown by red lines. The experimental and simulated traces overlap well, validating the results of the Tikhonov-based analysis.

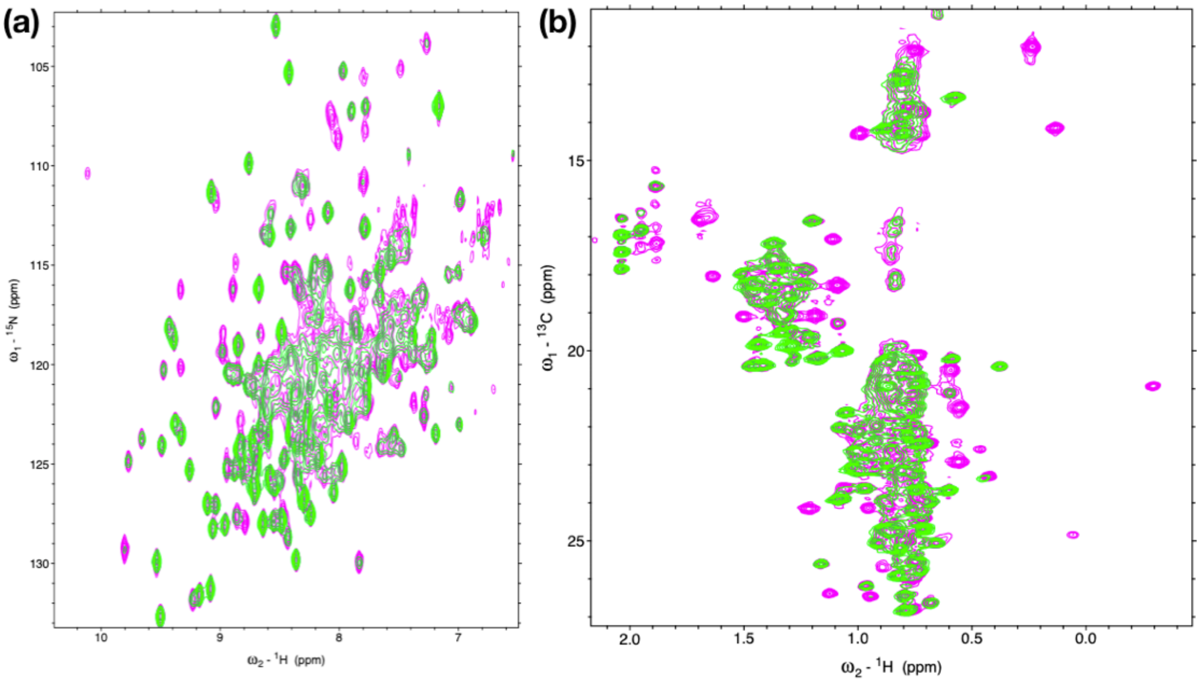

Figure S3. Paramagnetic relaxation enhancement (PRE) analysis of substrate recognition by full-length TF. The backbone ^{15}N-^{1}H TROSY (a) and side-chain methyl ^{13}C-^{1}H SOFAST-HMQC of U-[^{15}N,^{2}H], Ile-[δ1-$^{13}C_m$,$^{1}H_m$], [Leu/Val-[$^{13}C_m$,$^{1}H_m$], Ala-[β-$^{13}C_m$,$^{1}H_m$], Met-[^{13}C,^{1}H] PPI+SBD with oxidized and reduced MTSL-labeled IcdH3 peptide are shown in magenta and green, respectively.
